# Crystal structure of 2-(2,3-di­methyl­anilino)-*N*′-[(1*E*)-2-hy­droxy­benzyl­idene]benzohydrazide

**DOI:** 10.1107/S2056989015021532

**Published:** 2015-11-21

**Authors:** Shaaban K. Mohamed, Joel T. Mague, Mehmet Akkurt, Alaa F. Mohamed, Mustafa R. Albayati

**Affiliations:** a Faculty of Science & Engineering, School of Healthcare Science, Manchester Metropolitan University, M1 5GD, England; b Chemistry Department, Faculty of Science, Minia University, 61519 El-Minia, Egypt; c Department of Chemistry, Tulane University, New Orleans, LA 70118, USA; d Department of Physics, Faculty of Sciences, Erciyes University, 38039 Kayseri, Turkey; e National Organization for Drug Control and Research, Giza, Egypt; f Kirkuk University, College of Science, Department of Chemistry, Kirkuk, Iraq

**Keywords:** crystal structure, non-steroidal anti-inflammatory drugs (NSAIDs) mefenamic acid (MA), hydrazide-hydrazone compounds, hydrogen bonding

## Abstract

The asymmetric unit of the title compound, C_22_H_21_N_3_O_2_, consists of two independent mol­ecules (*A* and *B*) having differing conformations. The differences mainly concern the dihedral angles which the hy­droxy­phenyl and di­methyl­phenyl rings subtend to the central phenyl­ene ring, these being 30.16 (6) and 58.60 (6)° in mol­ecule *A* and 13.42 (7) and 60.31 (7)° in *B*. With the exception of the dimethyphenyl substituent, the conformations of the rest of each mol­ecule are largely determined by intra­molecular O—H⋯N and N—H⋯O hydrogen bonds. In the crystal, N—H⋯O hydrogen bonds link the mol­ecules into chains extending parallel to the *a* axis in which the types of mol­ecules alternate in an …*A*…*B*…*A*…*B*… fashion.

## Related literature   

For the medicinal use of mefenamic acid (MA), see: Nawaz *et al.* (2007 ▸); Joo *et al.* (2006 ▸). For the effects of masking the free acidic group in MA and other NSAIDs, see: Arun & Ashok (2009 ▸); Tammara *et al.* (1994 ▸). For various biological activities of hydrazide-hydrazone compounds, see: Bedia *et al.* (2006 ▸); Rollas *et al.* (2002 ▸); Palaska *et al.* (2002 ▸); Rollas & Küçükgüzel (2007 ▸).
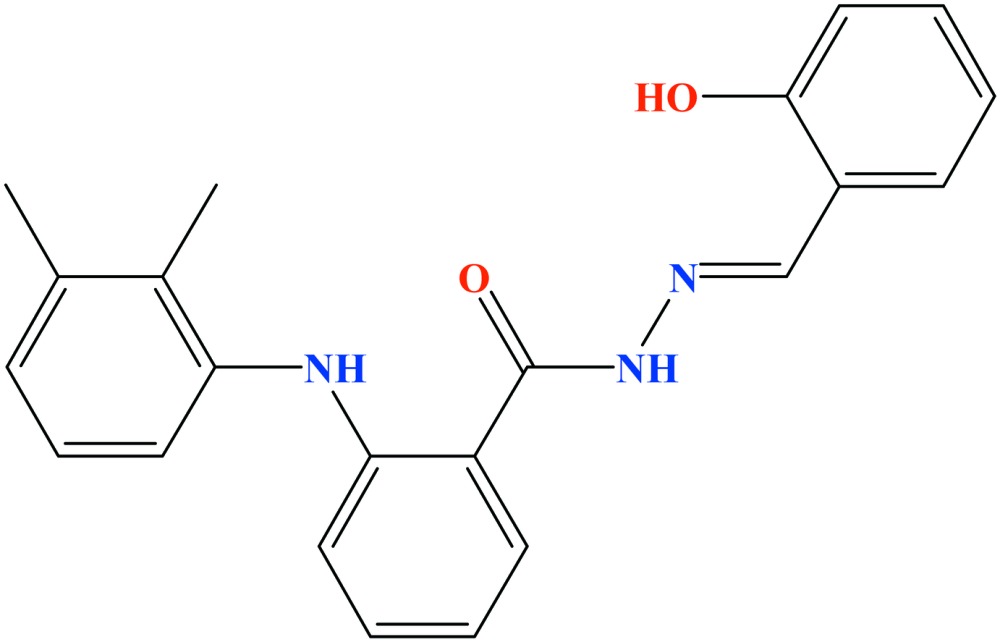


## Experimental   

### Crystal data  

C_22_H_21_N_3_O_2_
*M* *_r_* = 359.42
Monoclinic, 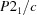

*a* = 10.8056 (8) Å
*b* = 14.7141 (12) Å
*c* = 23.0408 (18) Å
β = 96.181 (1)°
*V* = 3642.1 (5) Å^3^
*Z* = 8
Mo *K*α radiation
μ = 0.09 mm^−1^
*T* = 150 K
0.24 × 0.16 × 0.11 mm

### Data collection  

Bruker SMART APEX CCD diffractometer
Absorption correction: multi-scan (*SADABS*; Bruker, 2015 ▸) *T*_min_ = 0.86, *T*_max_ = 0.99
70656 measured reflections
10033 independent reflections
6949 reflections with *I* > 2σ(*I*)
*R*_int_ = 0.050

### Refinement  

*R*[*F* ^2^ > 2σ(*F* ^2^)] = 0.047
*wR*(*F* ^2^) = 0.133
*S* = 1.06
10033 reflections
515 parameters
6 restraints
H atoms treated by a mixture of independent and constrained refinement
Δρ_max_ = 0.32 e Å^−3^
Δρ_min_ = −0.20 e Å^−3^

### 

Data collection: *APEX2* (Bruker, 2015 ▸); cell refinement: *SAINT* (Bruker, 2015 ▸); data reduction: *SAINT*; program(s) used to solve structure: *SHELXS2014* (Sheldrick, 2008 ▸); program(s) used to refine structure: *SHELXL2014* (Sheldrick, 2015 ▸); molecular graphics: *ORTEP-3 for Windows* (Farrugia, 2012 ▸); software used to prepare material for publication: *PLATON* (Spek, 2009 ▸).

## Supplementary Material

Crystal structure: contains datablock(s) global, I. DOI: 10.1107/S2056989015021532/bg2574sup1.cif

Structure factors: contains datablock(s) I. DOI: 10.1107/S2056989015021532/bg2574Isup2.hkl

Supporting information file. DOI: 10.1107/S2056989015021532/bg2574Isup3.cml

**Fig. 1.**
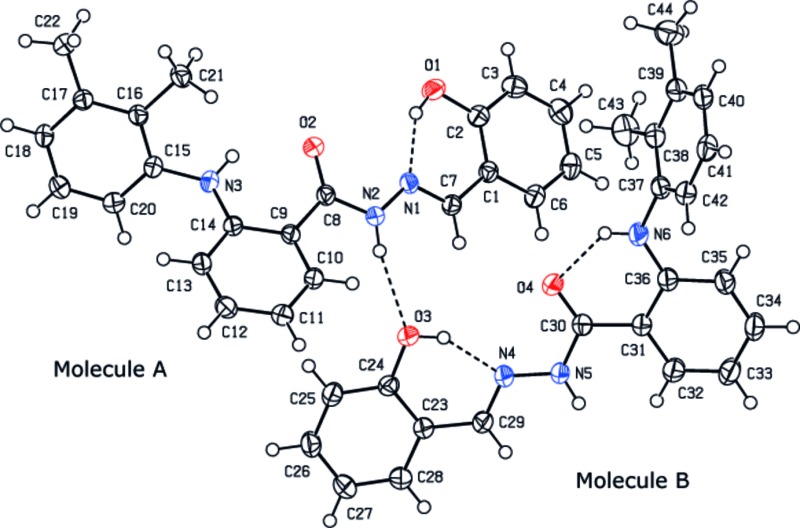
The asymmetric unit with labeling scheme and 50% probability ellipsoids. O—H···N and N—H···O hydrogen bonds are shown.

. DOI: 10.1107/S2056989015021532/bg2574fig1.tif
The asymmetric unit with labeling scheme and 50% probability ellipsoids. O—H⋯N and N—H⋯O hydrogen bonds are shown.

**Fig. 2.**
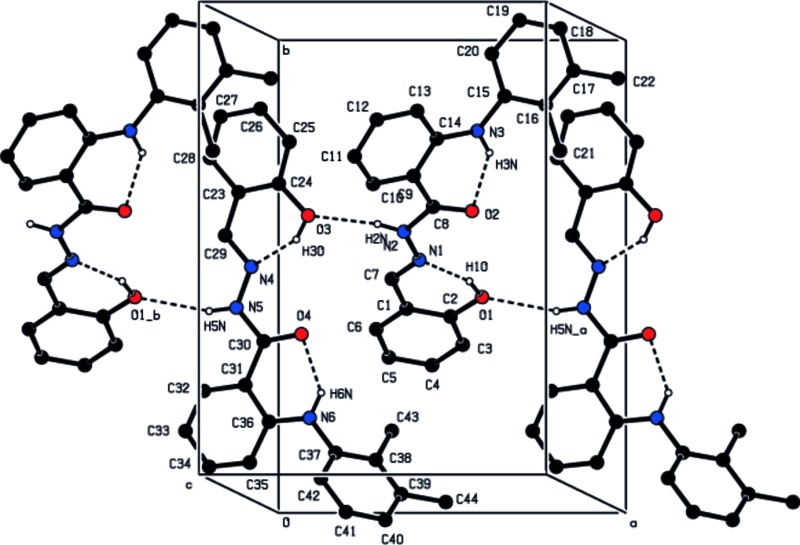
The cell-packing diagram of the title compound viewed down the c axis. Symmetry codes: (a) 1 + x, y, z; (b) 2 - x, -1/2 + y, 3/2 - z.

Click here for additional data file.c a x y z b x y z . DOI: 10.1107/S2056989015021532/bg2574fig2.tif
The cell-packing diagram of the title compound viewed down the *c* axis. Symmetry codes: (*a*) 1 + *x*, *y*, *z*; (*b*) 2 − *x*, −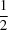 + *y*, 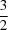 − *z*.

CCDC reference: 1436917

Additional supporting information:  crystallographic information; 3D view; checkCIF report

## Figures and Tables

**Table 1:** Hydrogen-bond geometry (Å, °)

*D*—H⋯*A*	*D*—H	H⋯*A*	*D*⋯*A*	*D*—H⋯*A*
O1—H1*O*⋯N1	0.87 (2)	1.83 (2)	2.6048 (14)	149 (2)
N2—H2*N*⋯O3	0.86 (1)	2.22 (1)	3.0640 (14)	167 (1)
N3—H3*N*⋯O2	0.87 (2)	2.01 (1)	2.7114 (15)	138 (1)
O3—H3*O*⋯N4	0.87 (2)	1.81 (2)	2.5854 (14)	149 (2)
N5—H5*N*⋯O1^i^	0.87 (1)	2.38 (1)	3.2164 (14)	162 (1)
N6—H6*N*⋯O4	0.86 (1)	1.98 (1)	2.6675 (15)	136 (1)

Symmetry code: (i) 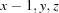.

## supplementary crystallographic information

### S1. Comment

The antiphlogistic non-steroidal anti-inflammatory drugs (NSAIDs) mefenamic acid [2-(2,3-dimethylphenylamino)-benzoic acid], is a potent *cyclo*-oxygenase inhibitor (Nawaz *et al.*, 2007). Mefenamic (MA) acid also has therapeutic potential in Alzheimer's disease (Joo *et al.*, 2006). Masking the free carboxylic group in MA like other NSAIDs could suppress the side effect such as gastrointestinal toxicity (Arun & Ashok, 2009; Tammara *et al.*, 1994). On other hand, hydrazide-hydrazone compounds are found to be associated with various biological activities such as antimicrobial, anti-convulsant, analgesic, anti-inflammatory, anti-platelet, anti-tubercular and anti-tumor properties (Bedia *et al.*, 2006; Rollas *et al.*, 2002; Palaska *et al.*, 2002; Rollas & Küçükgüzel, 2007). Based on such facts, and as part of our ongoing study on the functionalization of (NSAIDs), we report herein the synthesis and the crystal structure of the title compound.

The asymmetric unit of the title compound consists of two independent molecules (A and B) having differing conformations. Differences reside mainly in the dihedral angles which the hydroxyphenyl and dimethylphenyl rings make with the central phenylene ring, being 30.16 (6) and 58.60 (6)° in one of the molecules (A) and 13.42 (7) and 60.31 (7)° in the remaining one (B). With the exception of the dimethyphenyl substituent, the conformations of the rest of each molecule are largely determined by intramolecular O—H···N and N—H···O hydrogen bonds (Fig. 1 and Table 1). Intermolecular N—H···O hydrogen bonds form chains extending parallel to the *a* axis (Fig. 2 and Table 1) where molecules on both types alternate in a.. A···B···A···B.. fashion.

### S2. Experimental

A mixture of equimolar ratio of Mefenamic acid hydrazide (1 mmol, 255 mg) and salicaldehyde (1 mmol, 122 mg) with catalytic amount of glacial acetic acid was refluxed for 5 h. On cooling, the precipitate was separated then collected and recrystallized from ethanol to furnish the title compound as brown crystals with m.p= 457–459 K.

### S3. Refinement

H-atoms attached to carbon were placed in calculated positions (C—H = 0.95 - 0.98 Å) and included as riding contributions with isotropic displacement parameters 1.2 - 1.5 times those of the attached atoms. H-atoms attached to nitrogen and to oxygen were placed in locations derived from a difference map and refined freely. In order to adjust distances of hydrogen atoms of the NH and OH groups *DFIX* instruction was used with the target value of 0.85 (2) Å (O1—H1O, N2—H2N, N3—H3N, O3—H3O, N5—H5N and N6—H6N).

### Crystal data

**Table:**

C_22_H_21_N_3_O_2_	*F*(000) = 1520
*M**_r_* = 359.42	*D*_x_ = 1.311 Mg m^−^^3^
Monoclinic, *P*2_1_/*c*	Mo *K*α radiation, λ = 0.71073 Å
Hall symbol: -P 2ybc	Cell parameters from 9899 reflections
*a* = 10.8056 (8) Å	θ = 2.4–28.5°
*b* = 14.7141 (12) Å	µ = 0.09 mm^−^^1^
*c* = 23.0408 (18) Å	*T* = 150 K
β = 96.181 (1)°	Parallelepiped, brown
*V* = 3642.1 (5) Å^3^	0.24 × 0.16 × 0.11 mm
*Z* = 8	

### Data collection

**Table:**

Bruker SMART APEX CCD diffractometer	10033 independent reflections
Radiation source: fine-focus sealed tube	6949 reflections with *I* > 2σ(*I*)
Graphite monochromator	*R*_int_ = 0.050
Detector resolution: 8.3333 pixels mm^-1^	θ_max_ = 29.6°, θ_min_ = 1.6°
φ and ω scans	*h* = −14→15
Absorption correction: multi-scan (*SADABS*; Bruker, 2015)	*k* = −20→20
*T*_min_ = 0.86, *T*_max_ = 0.99	*l* = −31→31
70656 measured reflections	

### Refinement

**Table:**

Refinement on *F*^2^	6 restraints
Least-squares matrix: full	Hydrogen site location: mixed
*R*[*F*^2^ > 2σ(*F*^2^)] = 0.047	H atoms treated by a mixture of independent and constrained refinement
*wR*(*F*^2^) = 0.133	*w* = 1/[σ^2^(*F*_o_^2^) + (0.0614*P*)^2^ + 0.5126*P*] where *P* = (*F*_o_^2^ + 2*F*_c_^2^)/3
*S* = 1.06	(Δ/σ)_max_ = 0.001
10033 reflections	Δρ_max_ = 0.32 e Å^−^^3^
515 parameters	Δρ_min_ = −0.20 e Å^−^^3^

### Special details

**Table:**

Geometry. Bond distances, angles *etc*. have been calculated using the rounded fractional coordinates. All su's are estimated from the variances of the (full) variance-covariance matrix. The cell e.s.d.'s are taken into account in the estimation of distances, angles and torsion angles
Refinement. Refinement on *F*^2^ for ALL reflections except those flagged by the user for potential systematic errors. Weighted *R*-factors *wR* and all goodnesses of fit *S* are based on *F*^2^, conventional *R*-factors *R* are based on *F*, with *F* set to zero for negative *F*^2^. The observed criterion of *F*^2^ > σ(*F*^2^) is used only for calculating -*R*-factor-obs *etc*. and is not relevant to the choice of reflections for refinement. *R*-factors based on *F*^2^ are statistically about twice as large as those based on *F*, and *R*-factors based on ALL data will be even larger.

### Fractional atomic coordinates and isotropic or equivalent isotropic displacement parameters (Å^2^)

**Table:**

	*x*	*y*	*z*	*U*_iso_*/*U*_eq_	
O1	0.71230 (8)	0.41170 (7)	0.55128 (4)	0.0340 (3)	
O2	0.71178 (8)	0.58379 (6)	0.67590 (4)	0.0308 (3)	
N1	0.54495 (10)	0.48567 (7)	0.60962 (4)	0.0251 (3)	
N2	0.51064 (10)	0.54194 (7)	0.65258 (5)	0.0255 (3)	
N3	0.73926 (11)	0.74914 (8)	0.72762 (6)	0.0365 (4)	
C1	0.48962 (11)	0.38916 (8)	0.52842 (5)	0.0245 (3)	
C2	0.61366 (12)	0.37317 (9)	0.51773 (5)	0.0270 (3)	
C3	0.63844 (13)	0.31751 (10)	0.47176 (6)	0.0330 (4)	
O3	0.22998 (8)	0.57785 (6)	0.63251 (5)	0.0340 (3)	
C4	0.54195 (14)	0.27694 (10)	0.43666 (6)	0.0352 (4)	
O4	0.20203 (8)	0.32974 (6)	0.59410 (5)	0.0357 (3)	
C5	0.41977 (14)	0.29267 (9)	0.44609 (6)	0.0336 (4)	
C6	0.39397 (12)	0.34867 (9)	0.49152 (6)	0.0293 (3)	
C7	0.45795 (12)	0.44811 (8)	0.57523 (5)	0.0247 (3)	
C8	0.60152 (11)	0.59197 (8)	0.68416 (5)	0.0245 (3)	
C9	0.55567 (11)	0.65385 (8)	0.72794 (5)	0.0241 (3)	
C10	0.44464 (11)	0.63492 (8)	0.75139 (6)	0.0271 (3)	
C11	0.40069 (13)	0.68983 (9)	0.79312 (6)	0.0307 (4)	
C12	0.46933 (12)	0.76582 (9)	0.81250 (6)	0.0312 (4)	
C13	0.58055 (12)	0.78611 (9)	0.79129 (6)	0.0304 (4)	
C14	0.62745 (11)	0.73073 (8)	0.74880 (6)	0.0263 (4)	
C15	0.82217 (12)	0.82105 (9)	0.74591 (6)	0.0297 (4)	
C16	0.94530 (11)	0.80069 (8)	0.76726 (6)	0.0256 (3)	
C17	1.02858 (11)	0.87235 (8)	0.78144 (5)	0.0260 (3)	
C18	0.98854 (12)	0.96196 (8)	0.77383 (6)	0.0294 (4)	
C19	0.86683 (13)	0.98111 (9)	0.75292 (6)	0.0346 (4)	
C20	0.78358 (13)	0.91125 (9)	0.73913 (7)	0.0351 (4)	
C21	0.98772 (13)	0.70311 (9)	0.77297 (6)	0.0325 (4)	
C22	1.16205 (12)	0.85380 (10)	0.80475 (6)	0.0335 (4)	
N4	0.05545 (10)	0.47307 (7)	0.58508 (5)	0.0281 (3)	
N5	0.00971 (10)	0.38778 (7)	0.57123 (5)	0.0304 (3)	
N6	0.22941 (11)	0.15107 (8)	0.61035 (6)	0.0385 (4)	
C23	0.02226 (12)	0.62982 (9)	0.59976 (6)	0.0293 (4)	
C24	0.14408 (12)	0.64560 (8)	0.62567 (6)	0.0266 (3)	
C25	0.17876 (13)	0.73246 (9)	0.64497 (6)	0.0320 (4)	
C26	0.09406 (14)	0.80314 (9)	0.63855 (6)	0.0350 (4)	
C27	−0.02636 (14)	0.78881 (10)	0.61347 (7)	0.0410 (5)	
C28	−0.06137 (14)	0.70255 (10)	0.59448 (7)	0.0398 (4)	
C29	−0.02069 (12)	0.53978 (9)	0.58113 (6)	0.0324 (4)	
C30	0.09011 (12)	0.31571 (8)	0.58057 (6)	0.0274 (3)	
C31	0.03505 (12)	0.22381 (8)	0.57255 (6)	0.0280 (4)	
C32	−0.08981 (13)	0.21319 (10)	0.54979 (7)	0.0405 (5)	
C33	−0.14415 (15)	0.12908 (10)	0.54095 (8)	0.0483 (5)	
C34	−0.07282 (14)	0.05222 (10)	0.55509 (7)	0.0421 (5)	
C35	0.04937 (13)	0.05954 (9)	0.57768 (6)	0.0364 (4)	
C36	0.10719 (13)	0.14491 (9)	0.58750 (6)	0.0302 (4)	
C37	0.30784 (12)	0.07592 (9)	0.62829 (6)	0.0307 (4)	
C38	0.41823 (13)	0.06300 (9)	0.60272 (6)	0.0321 (4)	
C39	0.49524 (13)	−0.01087 (9)	0.62108 (6)	0.0331 (4)	
C40	0.45940 (13)	−0.06923 (9)	0.66372 (6)	0.0344 (4)	
C41	0.35190 (13)	−0.05417 (9)	0.68957 (6)	0.0347 (4)	
C42	0.27609 (13)	0.01856 (9)	0.67221 (6)	0.0339 (4)	
C43	0.45350 (17)	0.12825 (11)	0.55684 (7)	0.0479 (5)	
C44	0.61826 (15)	−0.02580 (12)	0.59715 (8)	0.0481 (6)	
H1O	0.6817 (16)	0.4431 (12)	0.5782 (6)	0.059 (6)*	
H2N	0.4343 (9)	0.5582 (10)	0.6517 (6)	0.031 (4)*	
H3	0.72210	0.30720	0.46430	0.0400*	
H3N	0.7699 (15)	0.7042 (9)	0.7096 (7)	0.048 (5)*	
H4	0.56000	0.23790	0.40580	0.0420*	
H5	0.35390	0.26520	0.42160	0.0400*	
H6	0.30980	0.35980	0.49780	0.0350*	
H7	0.37320	0.45890	0.58040	0.0300*	
H10	0.39800	0.58270	0.73820	0.0330*	
H11	0.32470	0.67590	0.80840	0.0370*	
H12	0.43910	0.80450	0.84090	0.0370*	
H13	0.62620	0.83810	0.80550	0.0360*	
H18	1.04550	1.01030	0.78310	0.0350*	
H19	0.84030	1.04240	0.74800	0.0420*	
H20	0.69970	0.92460	0.72500	0.0420*	
H21A	1.01680	0.68310	0.73620	0.0490*	
H21B	0.91810	0.66480	0.78190	0.0490*	
H21C	1.05580	0.69810	0.80450	0.0490*	
H22A	1.20620	0.91150	0.81210	0.0500*	
H22B	1.20240	0.81850	0.77600	0.0500*	
H22C	1.16430	0.81930	0.84120	0.0500*	
H3O	0.1931 (16)	0.5282 (9)	0.6200 (8)	0.061 (6)*	
H5N	−0.0706 (9)	0.3809 (11)	0.5650 (7)	0.045 (5)*	
H6N	0.2625 (15)	0.2043 (8)	0.6108 (7)	0.049 (5)*	
H25	0.26100	0.74330	0.66260	0.0380*	
H26	0.11900	0.86230	0.65150	0.0420*	
H27	−0.08430	0.83760	0.60930	0.0490*	
H28	−0.14420	0.69240	0.57740	0.0480*	
H29	−0.10510	0.53060	0.56610	0.0390*	
H32	−0.13850	0.26590	0.54020	0.0490*	
H33	−0.22890	0.12370	0.52540	0.0580*	
H34	−0.10920	−0.00630	0.54900	0.0510*	
H35	0.09620	0.00590	0.58690	0.0440*	
H40	0.50980	−0.12040	0.67520	0.0410*	
H41	0.33000	−0.09390	0.71930	0.0420*	
H42	0.20240	0.02930	0.69030	0.0410*	
H43A	0.49940	0.17960	0.57580	0.0720*	
H43B	0.37800	0.15060	0.53390	0.0720*	
H43C	0.50600	0.09700	0.53100	0.0720*	
H44A	0.67550	0.02370	0.61000	0.0720*	
H44B	0.60530	−0.02690	0.55440	0.0720*	
H44C	0.65390	−0.08390	0.61150	0.0720*	

### Atomic displacement parameters (Å^2^)

**Table:**

	*U*^11^	*U*^22^	*U*^33^	*U*^12^	*U*^13^	*U*^23^
O1	0.0239 (5)	0.0414 (6)	0.0360 (5)	−0.0004 (4)	−0.0001 (4)	−0.0079 (4)
O2	0.0234 (4)	0.0294 (5)	0.0393 (5)	−0.0001 (4)	0.0018 (4)	−0.0064 (4)
N1	0.0270 (5)	0.0200 (5)	0.0278 (5)	−0.0004 (4)	0.0014 (4)	−0.0010 (4)
N2	0.0220 (5)	0.0229 (5)	0.0312 (6)	0.0003 (4)	0.0013 (4)	−0.0044 (4)
N3	0.0257 (6)	0.0301 (6)	0.0546 (8)	−0.0067 (5)	0.0081 (5)	−0.0154 (5)
C1	0.0261 (6)	0.0215 (6)	0.0254 (6)	−0.0014 (5)	0.0005 (5)	0.0023 (5)
C2	0.0270 (6)	0.0271 (6)	0.0263 (6)	0.0000 (5)	0.0003 (5)	0.0038 (5)
C3	0.0329 (7)	0.0369 (7)	0.0293 (7)	0.0049 (6)	0.0040 (6)	0.0006 (6)
O3	0.0225 (4)	0.0281 (5)	0.0505 (6)	0.0009 (4)	−0.0008 (4)	−0.0015 (4)
C4	0.0450 (8)	0.0338 (7)	0.0265 (6)	0.0025 (6)	0.0026 (6)	−0.0026 (5)
O4	0.0253 (5)	0.0272 (5)	0.0541 (6)	−0.0029 (4)	0.0024 (4)	0.0013 (4)
C5	0.0388 (8)	0.0317 (7)	0.0288 (7)	−0.0064 (6)	−0.0026 (6)	−0.0023 (5)
C6	0.0285 (6)	0.0285 (6)	0.0303 (6)	−0.0038 (5)	−0.0002 (5)	0.0006 (5)
C7	0.0235 (6)	0.0223 (6)	0.0279 (6)	−0.0014 (5)	0.0004 (5)	0.0012 (5)
C8	0.0246 (6)	0.0193 (5)	0.0287 (6)	−0.0002 (5)	−0.0015 (5)	0.0009 (5)
C9	0.0231 (6)	0.0196 (5)	0.0284 (6)	0.0016 (4)	−0.0025 (5)	0.0004 (5)
C10	0.0254 (6)	0.0228 (6)	0.0321 (6)	−0.0014 (5)	−0.0014 (5)	−0.0004 (5)
C11	0.0278 (6)	0.0278 (6)	0.0368 (7)	0.0009 (5)	0.0045 (6)	−0.0001 (5)
C12	0.0320 (7)	0.0275 (6)	0.0336 (7)	0.0038 (5)	0.0019 (6)	−0.0035 (5)
C13	0.0293 (6)	0.0234 (6)	0.0370 (7)	−0.0001 (5)	−0.0034 (6)	−0.0049 (5)
C14	0.0227 (6)	0.0221 (6)	0.0330 (7)	0.0006 (5)	−0.0024 (5)	−0.0008 (5)
C15	0.0255 (6)	0.0264 (6)	0.0373 (7)	−0.0047 (5)	0.0042 (5)	−0.0067 (5)
C16	0.0258 (6)	0.0223 (6)	0.0295 (6)	−0.0015 (5)	0.0065 (5)	−0.0016 (5)
C17	0.0247 (6)	0.0260 (6)	0.0279 (6)	−0.0018 (5)	0.0058 (5)	−0.0026 (5)
C18	0.0296 (7)	0.0223 (6)	0.0366 (7)	−0.0053 (5)	0.0047 (5)	−0.0027 (5)
C19	0.0350 (7)	0.0222 (6)	0.0462 (8)	0.0015 (5)	0.0026 (6)	0.0001 (6)
C20	0.0256 (6)	0.0294 (7)	0.0492 (8)	0.0019 (5)	−0.0015 (6)	−0.0034 (6)
C21	0.0339 (7)	0.0227 (6)	0.0421 (8)	−0.0003 (5)	0.0091 (6)	0.0003 (5)
C22	0.0252 (6)	0.0345 (7)	0.0406 (8)	−0.0020 (5)	0.0026 (6)	−0.0017 (6)
N4	0.0287 (6)	0.0216 (5)	0.0338 (6)	−0.0015 (4)	0.0019 (5)	−0.0009 (4)
N5	0.0252 (6)	0.0221 (5)	0.0428 (6)	−0.0019 (4)	−0.0015 (5)	−0.0024 (5)
N6	0.0308 (6)	0.0216 (6)	0.0602 (8)	−0.0008 (5)	−0.0077 (6)	0.0000 (5)
C23	0.0264 (6)	0.0244 (6)	0.0366 (7)	−0.0002 (5)	0.0013 (5)	−0.0011 (5)
C24	0.0239 (6)	0.0263 (6)	0.0297 (6)	−0.0012 (5)	0.0041 (5)	0.0013 (5)
C25	0.0300 (7)	0.0306 (7)	0.0352 (7)	−0.0063 (5)	0.0030 (6)	−0.0016 (6)
C26	0.0404 (8)	0.0242 (6)	0.0405 (8)	−0.0050 (6)	0.0054 (6)	−0.0021 (6)
C27	0.0395 (8)	0.0258 (7)	0.0568 (9)	0.0052 (6)	0.0012 (7)	−0.0010 (6)
C28	0.0296 (7)	0.0303 (7)	0.0570 (9)	0.0036 (6)	−0.0063 (7)	−0.0039 (7)
C29	0.0259 (6)	0.0273 (6)	0.0423 (8)	−0.0012 (5)	−0.0038 (6)	−0.0023 (6)
C30	0.0276 (6)	0.0244 (6)	0.0303 (6)	−0.0013 (5)	0.0037 (5)	0.0001 (5)
C31	0.0272 (6)	0.0237 (6)	0.0329 (7)	−0.0021 (5)	0.0017 (5)	−0.0015 (5)
C32	0.0315 (7)	0.0287 (7)	0.0588 (10)	−0.0009 (6)	−0.0060 (7)	0.0020 (7)
C33	0.0343 (8)	0.0338 (8)	0.0727 (11)	−0.0064 (6)	−0.0128 (8)	−0.0001 (8)
C34	0.0412 (8)	0.0269 (7)	0.0563 (9)	−0.0088 (6)	−0.0040 (7)	−0.0025 (7)
C35	0.0375 (8)	0.0233 (6)	0.0466 (8)	−0.0003 (6)	−0.0036 (6)	−0.0015 (6)
C36	0.0314 (7)	0.0253 (6)	0.0336 (7)	−0.0020 (5)	0.0020 (5)	−0.0012 (5)
C37	0.0303 (7)	0.0220 (6)	0.0379 (7)	0.0003 (5)	−0.0046 (6)	−0.0024 (5)
C38	0.0367 (7)	0.0262 (6)	0.0323 (7)	−0.0030 (5)	−0.0012 (6)	−0.0014 (5)
C39	0.0318 (7)	0.0289 (6)	0.0376 (7)	0.0000 (5)	−0.0007 (6)	−0.0064 (6)
C40	0.0342 (7)	0.0229 (6)	0.0435 (8)	−0.0009 (5)	−0.0081 (6)	−0.0014 (6)
C41	0.0374 (7)	0.0281 (7)	0.0372 (7)	−0.0080 (6)	−0.0024 (6)	0.0034 (6)
C42	0.0318 (7)	0.0282 (7)	0.0411 (8)	−0.0034 (5)	0.0017 (6)	−0.0013 (6)
C43	0.0606 (11)	0.0431 (9)	0.0401 (8)	−0.0037 (8)	0.0058 (8)	0.0062 (7)
C44	0.0424 (9)	0.0501 (10)	0.0523 (10)	0.0064 (7)	0.0081 (8)	−0.0063 (8)

### Geometric parameters (Å, º)

**Table:**

O1—C2	1.3703 (16)	C19—H19	0.9500
O2—C8	1.2325 (15)	C20—H20	0.9500
N1—N2	1.3718 (15)	C21—H21A	0.9800
N1—C7	1.2871 (16)	C21—H21C	0.9800
O1—H1O	0.867 (16)	C21—H21B	0.9800
N2—C8	1.3718 (16)	C22—H22A	0.9800
N3—C14	1.3777 (17)	C22—H22C	0.9800
N3—C15	1.4207 (18)	C22—H22B	0.9800
C1—C6	1.3989 (18)	N5—H5N	0.870 (10)
C1—C7	1.4534 (17)	N6—H6N	0.861 (13)
C1—C2	1.4084 (18)	C23—C29	1.4533 (19)
C2—C3	1.3876 (19)	C23—C24	1.4049 (19)
N2—H2N	0.857 (10)	C23—C28	1.398 (2)
N3—H3N	0.865 (15)	C24—C25	1.3913 (18)
C3—C4	1.384 (2)	C25—C26	1.383 (2)
O3—C24	1.3597 (15)	C26—C27	1.382 (2)
O4—C30	1.2334 (16)	C27—C28	1.382 (2)
C4—C5	1.380 (2)	C30—C31	1.4809 (17)
C5—C6	1.3839 (19)	C31—C36	1.4200 (18)
C8—C9	1.4831 (17)	C31—C32	1.403 (2)
C9—C14	1.4255 (17)	C32—C33	1.376 (2)
C9—C10	1.3959 (17)	C33—C34	1.388 (2)
C10—C11	1.3788 (19)	C34—C35	1.370 (2)
C11—C12	1.3887 (19)	C35—C36	1.4103 (19)
C12—C13	1.3780 (19)	C37—C38	1.3991 (19)
C13—C14	1.4093 (19)	C37—C42	1.3888 (19)
C15—C20	1.3949 (19)	C38—C43	1.507 (2)
C15—C16	1.4004 (18)	C38—C39	1.4061 (19)
C16—C21	1.5086 (18)	C39—C40	1.3909 (19)
C16—C17	1.4018 (17)	C39—C44	1.509 (2)
C17—C22	1.5087 (18)	C40—C41	1.379 (2)
C17—C18	1.3929 (17)	C41—C42	1.3805 (19)
C18—C19	1.3801 (19)	C25—H25	0.9500
C19—C20	1.3803 (19)	C26—H26	0.9500
C3—H3	0.9500	C27—H27	0.9500
O3—H3O	0.867 (15)	C28—H28	0.9500
C4—H4	0.9500	C29—H29	0.9500
N4—N5	1.3737 (15)	C32—H32	0.9500
N4—C29	1.2777 (17)	C33—H33	0.9500
N5—C30	1.3731 (16)	C34—H34	0.9500
C5—H5	0.9500	C35—H35	0.9500
N6—C36	1.3711 (19)	C40—H40	0.9500
N6—C37	1.4270 (18)	C41—H41	0.9500
C6—H6	0.9500	C42—H42	0.9500
C7—H7	0.9500	C43—H43A	0.9800
C10—H10	0.9500	C43—H43B	0.9800
C11—H11	0.9500	C43—H43C	0.9800
C12—H12	0.9500	C44—H44A	0.9800
C13—H13	0.9500	C44—H44B	0.9800
C18—H18	0.9500	C44—H44C	0.9800
			
N2—N1—C7	117.85 (11)	H22A—C22—H22B	109.00
C2—O1—H1O	106.9 (11)	H22A—C22—H22C	110.00
N1—N2—C8	118.23 (10)	C17—C22—H22B	109.00
C14—N3—C15	126.18 (12)	C17—C22—H22A	110.00
C2—C1—C6	118.51 (11)	C30—N5—H5N	122.6 (11)
C2—C1—C7	122.28 (11)	N4—N5—H5N	118.2 (11)
C6—C1—C7	119.19 (11)	C36—N6—H6N	116.5 (10)
O1—C2—C1	121.96 (11)	C37—N6—H6N	118.0 (10)
O1—C2—C3	118.20 (12)	C24—C23—C28	118.55 (12)
N1—N2—H2N	119.1 (9)	C28—C23—C29	119.30 (12)
C8—N2—H2N	119.7 (10)	C24—C23—C29	122.07 (12)
C1—C2—C3	119.84 (12)	O3—C24—C25	118.65 (12)
C15—N3—H3N	116.7 (10)	O3—C24—C23	121.65 (11)
C14—N3—H3N	114.5 (10)	C23—C24—C25	119.70 (12)
C2—C3—C4	120.36 (13)	C24—C25—C26	120.29 (13)
C3—C4—C5	120.58 (13)	C25—C26—C27	120.84 (13)
C4—C5—C6	119.53 (13)	C26—C27—C28	119.07 (14)
C1—C6—C5	121.16 (12)	C23—C28—C27	121.56 (14)
N1—C7—C1	119.89 (11)	N4—C29—C23	119.78 (12)
O2—C8—C9	124.35 (11)	O4—C30—C31	123.66 (11)
N2—C8—C9	114.69 (10)	N5—C30—C31	116.54 (11)
O2—C8—N2	120.96 (11)	O4—C30—N5	119.80 (11)
C8—C9—C14	120.47 (11)	C30—C31—C36	120.91 (12)
C10—C9—C14	119.14 (11)	C32—C31—C36	118.71 (12)
C8—C9—C10	120.31 (11)	C30—C31—C32	120.38 (12)
C9—C10—C11	121.87 (11)	C31—C32—C33	122.25 (13)
C10—C11—C12	118.94 (12)	C32—C33—C34	118.76 (15)
C11—C12—C13	121.09 (13)	C33—C34—C35	120.88 (14)
C12—C13—C14	120.91 (12)	C34—C35—C36	121.54 (13)
N3—C14—C9	120.18 (11)	N6—C36—C31	121.34 (12)
N3—C14—C13	121.80 (11)	N6—C36—C35	120.81 (12)
C9—C14—C13	118.02 (11)	C31—C36—C35	117.85 (12)
C16—C15—C20	120.28 (12)	C38—C37—C42	120.86 (12)
N3—C15—C20	120.27 (12)	N6—C37—C42	119.76 (12)
N3—C15—C16	119.33 (12)	N6—C37—C38	119.34 (12)
C15—C16—C21	120.17 (11)	C37—C38—C43	120.02 (13)
C15—C16—C17	118.87 (11)	C37—C38—C39	118.83 (12)
C17—C16—C21	120.94 (11)	C39—C38—C43	121.15 (13)
C16—C17—C18	119.99 (11)	C38—C39—C40	119.28 (13)
C16—C17—C22	120.80 (11)	C38—C39—C44	121.25 (13)
C18—C17—C22	119.22 (11)	C40—C39—C44	119.44 (13)
C17—C18—C19	120.57 (12)	C39—C40—C41	121.16 (13)
C18—C19—C20	120.08 (12)	C40—C41—C42	120.04 (13)
C15—C20—C19	120.21 (13)	C37—C42—C41	119.79 (13)
C2—C3—H3	120.00	C24—C25—H25	120.00
C4—C3—H3	120.00	C26—C25—H25	120.00
C24—O3—H3O	107.2 (11)	C25—C26—H26	120.00
C5—C4—H4	120.00	C27—C26—H26	120.00
C3—C4—H4	120.00	C26—C27—H27	120.00
N5—N4—C29	118.19 (11)	C28—C27—H27	120.00
C4—C5—H5	120.00	C23—C28—H28	119.00
C6—C5—H5	120.00	C27—C28—H28	119.00
N4—N5—C30	117.61 (10)	N4—C29—H29	120.00
C36—N6—C37	125.27 (12)	C23—C29—H29	120.00
C5—C6—H6	119.00	C31—C32—H32	119.00
C1—C6—H6	119.00	C33—C32—H32	119.00
N1—C7—H7	120.00	C32—C33—H33	121.00
C1—C7—H7	120.00	C34—C33—H33	121.00
C9—C10—H10	119.00	C33—C34—H34	120.00
C11—C10—H10	119.00	C35—C34—H34	120.00
C12—C11—H11	121.00	C34—C35—H35	119.00
C10—C11—H11	121.00	C36—C35—H35	119.00
C11—C12—H12	119.00	C39—C40—H40	119.00
C13—C12—H12	119.00	C41—C40—H40	119.00
C14—C13—H13	120.00	C40—C41—H41	120.00
C12—C13—H13	120.00	C42—C41—H41	120.00
C17—C18—H18	120.00	C37—C42—H42	120.00
C19—C18—H18	120.00	C41—C42—H42	120.00
C20—C19—H19	120.00	C38—C43—H43A	109.00
C18—C19—H19	120.00	C38—C43—H43B	109.00
C19—C20—H20	120.00	C38—C43—H43C	110.00
C15—C20—H20	120.00	H43A—C43—H43B	109.00
C16—C21—H21B	109.00	H43A—C43—H43C	110.00
H21A—C21—H21C	109.00	H43B—C43—H43C	109.00
C16—C21—H21C	109.00	C39—C44—H44A	109.00
C16—C21—H21A	109.00	C39—C44—H44B	109.00
H21A—C21—H21B	109.00	C39—C44—H44C	109.00
H21B—C21—H21C	109.00	H44A—C44—H44B	109.00
C17—C22—H22C	109.00	H44A—C44—H44C	110.00
H22B—C22—H22C	109.00	H44B—C44—H44C	109.00
			
C7—N1—N2—C8	−171.12 (11)	C29—N4—N5—C30	−174.36 (12)
N2—N1—C7—C1	178.96 (10)	N5—N4—C29—C23	175.18 (12)
N1—N2—C8—O2	−3.03 (17)	N4—N5—C30—O4	−8.56 (19)
N1—N2—C8—C9	177.51 (10)	N4—N5—C30—C31	172.40 (11)
C15—N3—C14—C9	−177.55 (12)	C37—N6—C36—C31	177.96 (13)
C15—N3—C14—C13	2.3 (2)	C37—N6—C36—C35	−2.5 (2)
C14—N3—C15—C16	122.74 (15)	C36—N6—C37—C38	121.90 (15)
C14—N3—C15—C20	−61.4 (2)	C36—N6—C37—C42	−60.4 (2)
C6—C1—C2—O1	178.81 (12)	C28—C23—C24—O3	179.60 (13)
C6—C1—C2—C3	−0.66 (18)	C28—C23—C24—C25	−0.4 (2)
C7—C1—C2—O1	0.62 (19)	C29—C23—C24—O3	2.8 (2)
C7—C1—C2—C3	−178.85 (12)	C29—C23—C24—C25	−177.26 (13)
C2—C1—C6—C5	1.21 (19)	C24—C23—C28—C27	0.7 (2)
C7—C1—C6—C5	179.46 (12)	C29—C23—C28—C27	177.66 (14)
C2—C1—C7—N1	−1.99 (18)	C24—C23—C29—N4	−4.1 (2)
C6—C1—C7—N1	179.83 (12)	C28—C23—C29—N4	179.10 (13)
O1—C2—C3—C4	179.92 (12)	O3—C24—C25—C26	179.76 (13)
C1—C2—C3—C4	−0.6 (2)	C23—C24—C25—C26	−0.2 (2)
C2—C3—C4—C5	1.3 (2)	C24—C25—C26—C27	0.6 (2)
C3—C4—C5—C6	−0.8 (2)	C25—C26—C27—C28	−0.3 (2)
C4—C5—C6—C1	−0.5 (2)	C26—C27—C28—C23	−0.4 (2)
O2—C8—C9—C10	−154.06 (12)	O4—C30—C31—C32	−171.61 (14)
O2—C8—C9—C14	22.71 (18)	O4—C30—C31—C36	8.2 (2)
N2—C8—C9—C10	25.38 (16)	N5—C30—C31—C32	7.39 (19)
N2—C8—C9—C14	−157.85 (11)	N5—C30—C31—C36	−172.78 (12)
C8—C9—C10—C11	178.52 (12)	C30—C31—C32—C33	179.16 (15)
C14—C9—C10—C11	1.71 (19)	C36—C31—C32—C33	−0.7 (2)
C8—C9—C14—N3	1.17 (18)	C30—C31—C36—N6	0.7 (2)
C8—C9—C14—C13	−178.69 (11)	C30—C31—C36—C35	−178.83 (13)
C10—C9—C14—N3	177.98 (12)	C32—C31—C36—N6	−179.48 (14)
C10—C9—C14—C13	−1.89 (18)	C32—C31—C36—C35	1.0 (2)
C9—C10—C11—C12	−0.3 (2)	C31—C32—C33—C34	0.0 (3)
C10—C11—C12—C13	−0.9 (2)	C32—C33—C34—C35	0.3 (3)
C11—C12—C13—C14	0.6 (2)	C33—C34—C35—C36	0.1 (2)
C12—C13—C14—N3	−179.10 (13)	C34—C35—C36—N6	179.74 (14)
C12—C13—C14—C9	0.77 (19)	C34—C35—C36—C31	−0.7 (2)
N3—C15—C16—C17	175.69 (12)	N6—C37—C38—C39	179.50 (12)
N3—C15—C16—C21	−2.4 (2)	N6—C37—C38—C43	0.2 (2)
C20—C15—C16—C17	−0.2 (2)	C42—C37—C38—C39	1.8 (2)
C20—C15—C16—C21	−178.30 (13)	C42—C37—C38—C43	−177.53 (13)
N3—C15—C20—C19	−175.23 (14)	N6—C37—C42—C41	179.98 (12)
C16—C15—C20—C19	0.6 (2)	C38—C37—C42—C41	−2.3 (2)
C15—C16—C17—C18	−0.44 (19)	C37—C38—C39—C40	0.4 (2)
C15—C16—C17—C22	179.82 (12)	C37—C38—C39—C44	−177.13 (13)
C21—C16—C17—C18	177.67 (12)	C43—C38—C39—C40	179.73 (13)
C21—C16—C17—C22	−2.07 (19)	C43—C38—C39—C44	2.2 (2)
C16—C17—C18—C19	0.65 (19)	C38—C39—C40—C41	−2.1 (2)
C22—C17—C18—C19	−179.61 (12)	C44—C39—C40—C41	175.46 (14)
C17—C18—C19—C20	−0.2 (2)	C39—C40—C41—C42	1.6 (2)
C18—C19—C20—C15	−0.4 (2)	C40—C41—C42—C37	0.6 (2)

### Hydrogen-bond geometry (Å, º)

**Table:**

*D*—H···*A*	*D*—H	H···*A*	*D*···*A*	*D*—H···*A*
O1—H1*O*···N1	0.87 (2)	1.83 (2)	2.6048 (14)	149 (2)
N2—H2*N*···O3	0.86 (1)	2.22 (1)	3.0640 (14)	167 (1)
N3—H3*N*···O2	0.87 (2)	2.01 (1)	2.7114 (15)	138 (1)
O3—H3*O*···N4	0.87 (2)	1.81 (2)	2.5854 (14)	149 (2)
N5—H5*N*···O1^i^	0.87 (1)	2.38 (1)	3.2164 (14)	162 (1)
N6—H6*N*···O4	0.86 (1)	1.98 (1)	2.6675 (15)	136 (1)
C21—H21*B*···N3	0.98	2.51	2.8556 (19)	100
C32—H32···N5	0.95	2.46	2.8081 (18)	102
C43—H43*B*···N6	0.98	2.51	2.853 (2)	100

Symmetry code: (i) *x*−1, *y*, *z*.
