# Supplementary figures and images for: Crystal structure of 2-(2,3-di­methyl­anilino)-N′-[(1E)-2-hy­droxy­benzyl­idene]benzohydrazide
Source: Acta Crystallogr E Crystallogr Commun. 2015 Nov 21;71(Pt 12):o957–8. doi: 10.1107/S2056989015021532 (PMC4719921; doi:10.1107/S2056989015021532)

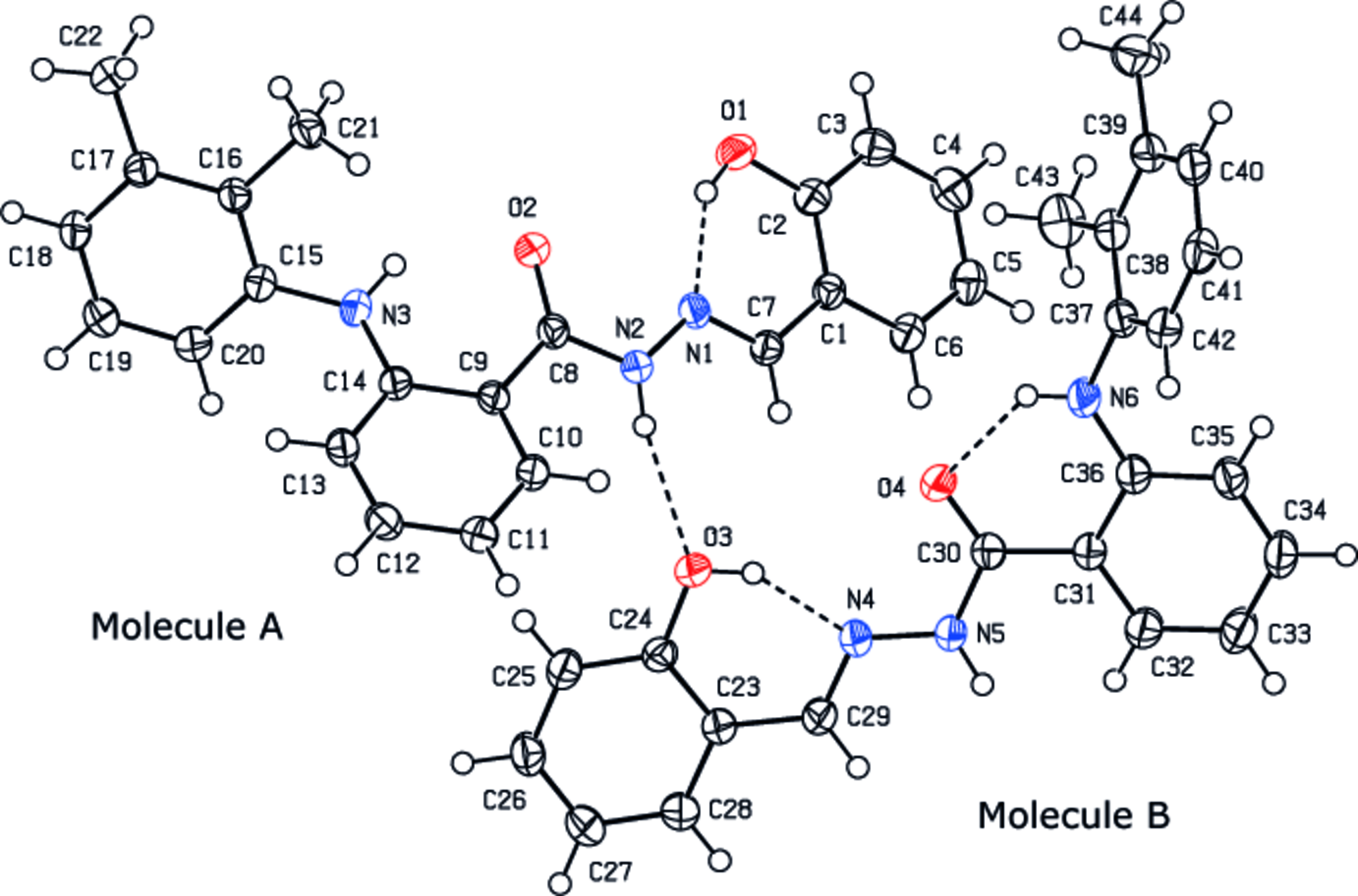

Supplement: Supplementary file 4 [file e-71-0o957-fig1.tif]

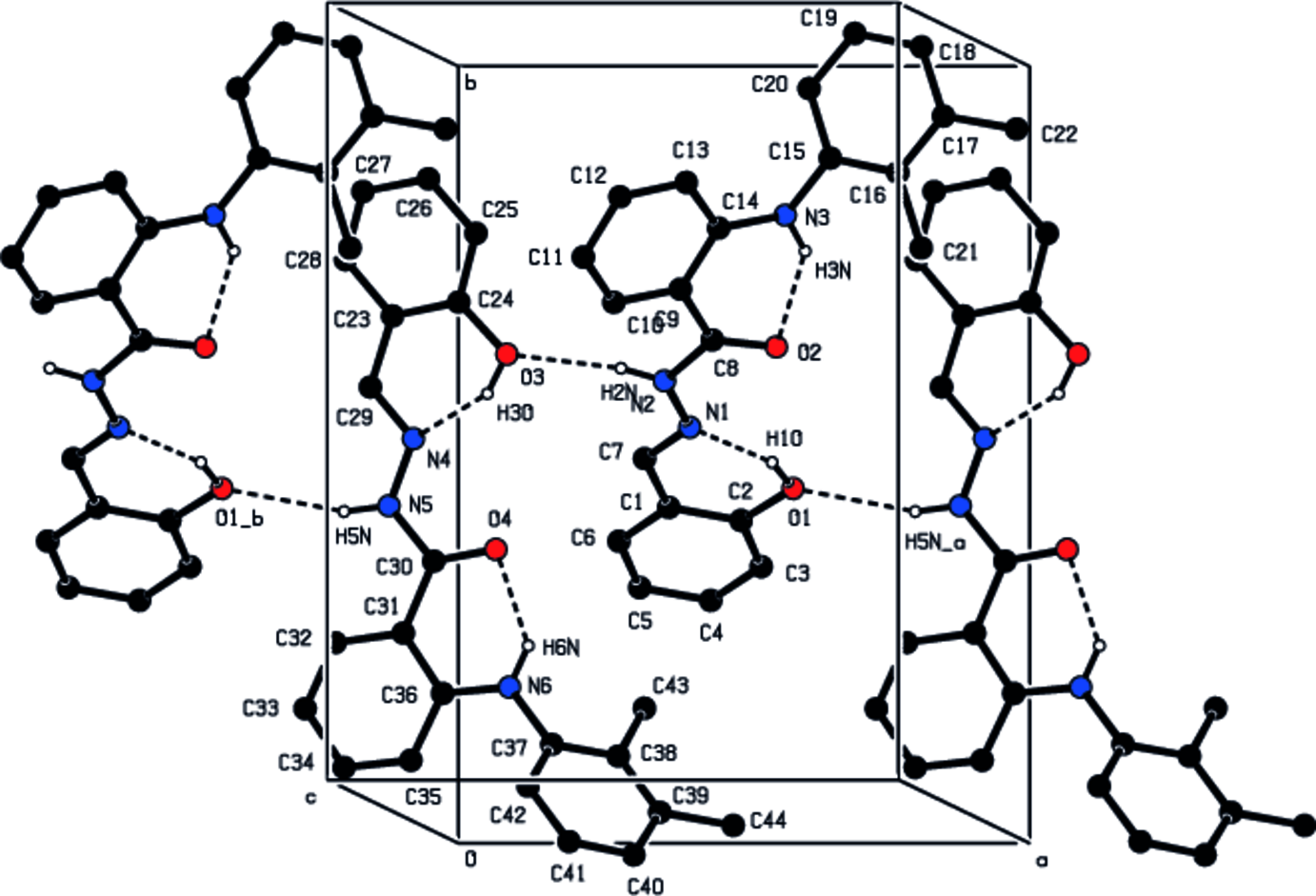

Supplement: Supplementary file 5 [file e-71-0o957-fig2.tif]
